# The Fate of Microplastics, Derived from Disposable Masks, in Natural Aquatic Environments

**DOI:** 10.3390/toxics12010061

**Published:** 2024-01-12

**Authors:** Wei Zhang, Senyou Chai, Changhui Duan, Xueliang Sun, Qiting Zuo, Lin Gong

**Affiliations:** 1 School of Ecology and Environment, Zhengzhou University, 100 Kexue Avenue, Zhengzhou 450001, China; zhangwei88@zzu.edu.cn (W.Z.); 19903887284@163.com (S.C.); 2 Henan International Joint Laboratory of Water Cycle Simulation and Environmental Protection, Zhengzhou 450001, China; zuoqt@zzu.edu.cn; 3 Henan Key Laboratory of Ecological Environment Protection and Restoration of Yellow River Basin, Zhengzhou 450000, China; 4 Yellow River Institute for Ecological Protection and Regional Coordination Development, Zhengzhou University, 100 Kexue Avenue, Zhengzhou 450001, China; 5 Henan Key Laboratory of Water Pollution Control and Rehabilitation Technology, Pingdingshan 467036, China; 6 Henan Key Laboratory of Water Resources Conservation and Intensive Utilization in the Yellow River Basin, Zhengzhou 450001, China; 7 Changzhi City Urban River Affairs Center, Changzhi 046000, China; dch202424@163.com; 8 China Planning Institute (Beijing) Planning and Design Co., Beijing 100044, China; xueliangsun@126.com

**Keywords:** fate of microplastics, personal protective equipment, environmental impact, challenges for control of microplastic

## Abstract

This paper mainly reviews the fate of microplastics, released from used face masks, in the water environment. Through previous experiments, the amount of fiber microplastics released from used face masks into aqueous environments was not negligible, with the maximum microplastics releasing amount reaching 10,000 piece·day^−1^ for each mask. Microplastic derived from these masks often occurred in the shape of polymeric fibers that resulted from the breakage of the chemical bonds in the plastic fibers by the force of water flow. The potential contact forces between microplastics (originating from face masks) with other pollutants, primarily encompass hydrophobic and electrostatic interactions. This critical review paper briefly illustrates the fate of microplastics derived from disposable face masks, further devising effective strategies to mitigate the environmental impact of plastic particle release from the used personal protective equipment.

## 1. Introduction

Over the past three years, the global landscape has been significantly impacted by the widespread transmission of COVID-19 [1,2,3]. A consequential outcome of this pandemic has been the exponential surge in the production and utilization of face masks, a crucial preventive measure adopted by individuals and communities alike [2,3,4,5,6,7,8]. However, an unintended consequence of this widespread use is the inevitable increase in the number of masks being disposed of haphazardly, contributing to environmental pollution and posing multifaceted challenges [4,5,6,7,8,9,10]. In particular, the improper disposal of used masks poses a significant environmental threat, leading to the release of various pollutants into the natural environment. These pollutants encompass a spectrum of hazardous substances, including but not limited to heavy metals, organic compounds, microplastics, as well as viruses and bacteria [4,5,6,7,8,9,10]. The potential consequences of such pollution extend far beyond environmental aesthetics, reaching into the realms of human safety, soil and water quality, and even the well-being of aquatic biota, invertebrates, and vertebrates. The intricate web of interconnected ecosystems underscores the urgency of addressing this issue comprehensively. Of notable concern is the fact that used masks exhibit a propensity to adsorb antibiotic pollutants present in both freshwater and seawater, thereby intensifying their toxicity within the natural environment [11]. This underscores the need for heightened awareness and effective management strategies to handle the disposal of spent masks responsibly. As the world grapples with the ongoing challenges posed by COVID-19, it becomes imperative to not only focus on immediate health concerns but also to address the environmental ramifications associated with the increased usage and disposal of protective equipment.

In the context of the prevailing circumstances, it is imperative to strongly advocate for and actively implement robust management and treatment protocols specifically tailored for the disposal of used masks in the unprecedented era dominated by the COVID-19 pandemic. This imperative arises from the escalating challenges posed by the extensive use of masks, necessitating a strategic and conscientious approach towards their lifecycle, from production to post-utilization stages. Concurrently, a pertinent avenue for addressing the environmental conundrum associated with disposable masks lies in the exploration and promotion of reusable mask options. This alternative presents itself as a potential solution with multifaceted benefits, aligning seamlessly with sustainable practices and serving as a proactive measure to mitigate the far-reaching environmental impact attributed to the prevalent usage of single-use masks [12,13]. The pressing need for such strategic shifts in mask management practices is underscored by the intricate interplay between public health, environmental stewardship, and sustainable development goals. By championing the cause of reusable masks, there is an opportunity to not only reduce the accumulation of discarded masks in natural ecosystems but also to instigate positive ripple effects that resonate across diverse sectors.

**Figure 1:**
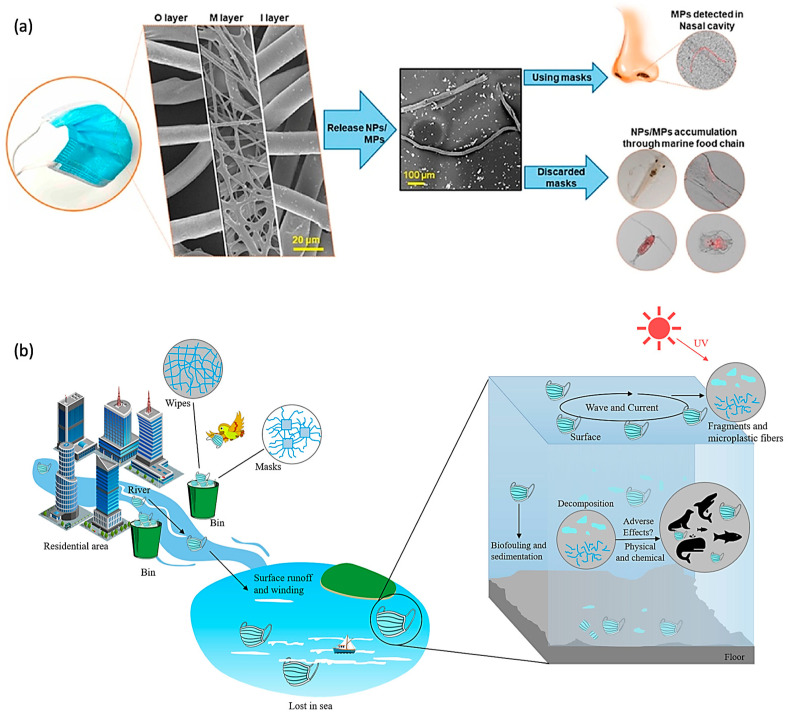
The proposed route for microplastics released from the masks and the potential threat to the body and outside environment: (**a**) Nano-plastics (NPs) and microplastics (MPs) released from face masks to the nasal cavity and marine food chain. (**b**) Waste masks generated from residential areas undergo various physical and chemical processes in the water environment, under the influence of some external environments, for example, UV (ultraviolet) light conditions. Adapted with permission from Ref. [21]. Copyright 2022, Springer/Adapted with permission from Ref. [22]. Copyright 2021, Elsevier.

In recent years, there has been a notable surge in research efforts dedicated to comprehensively understanding the intricate dynamics surrounding the generation, migration, and pollution of microplastics within the water and soil environments [14,15,16,17]. This heightened scientific inquiry is rooted in the recognition of the significant role played by microplastics in the environmental landscape. Microplastics, encompassing both micro-sized and nano-sized particles, have been identified as agents with a propensity to adsorb an array of pollutants, including heavy metals, organic pollutants, and various industrial chemicals [3,18,19]. Through the adsorption process, the microplastics not only contribute to an escalation in the toxicity of the absorbed pollutants but also serve as conduits for the transfer of these contaminants along the food chain, ultimately leading to their release into microorganisms, animals, and even human beings (Figure 1a,b). Consequently, recent research endeavors have focused on a specific facet of microplastic pollution, delving into the release and migration of microplastics derived from discarded masks [20]. This focused exploration is motivated by the growing concern surrounding the environmental impact of mask disposal and seeks to elucidate the potential of discarded masks to contribute to aquatic pollution. The unique characteristics of mask materials, coupled with their widespread usage and disposal patterns, necessitate a nuanced examination of the potential consequences for aquatic ecosystems. Through a holistic examination of the complexity of microplastic pollution derived from spent masks, society could work towards implementing informed policies and practices that strike a balance between public health measures and environmental conservation, thereby fostering a sustainable coexistence between human activities and the delicate ecosystems that surround us.

In this context, our investigation undertakes a comprehensive review, placing a primary emphasis on elucidating the processes involved in the generation of microplastics from masks and their subsequent release and migration within the aquatic environment.

Simultaneously, this article has provided a comprehensive review of the mutual processes and mechanisms between the released microplastics and potential hazardous substances in the environment.

Our research systematically searched nearly 300 publications in Web of Science and China CNKI databases, using keywords such as “microplastics”, “disposable Masks”, “personal protective equipment”, “environmental impact”, and “Challenges for control of microplastic”. We have selected 54 literature sources from the past 5 years for summarization. If cases where the viewpoints described in the literatures were similar, the journal literature with higher impact factors was prioritized.

## 2. Quantity of Microplastics Derived from the Masks into the Water Environment

In general, apart from the ear loop and the nose bridge, the main part of masks is usually made from polymeric fibers. The latter are derived from different types of plastics including polypropylene (PP), polyethylene terephthalate (PET), polyethylene (PE) and polyamide (PA), with the choice of the plastic being determined by the user type. The body of a mask is then commonly divided into three key layers, namely the inner layer, the middle layer and the outer layer, with plastic components being usually present in all of them [23]. The inner layer, in direct contact with the wearer’s face, is designed for optimal comfort, often featuring softer materials to prevent irritation. Meanwhile, the middle layer serves as a crucial filtration barrier, capturing particles and impurities, and is commonly reinforced with polymeric components to enhance its efficacy. The outer layer, exposed to the external environment, is crafted to provide durability and additional protection, with plastic elements contributing to its resilience against external factors.

**Figure 2:**
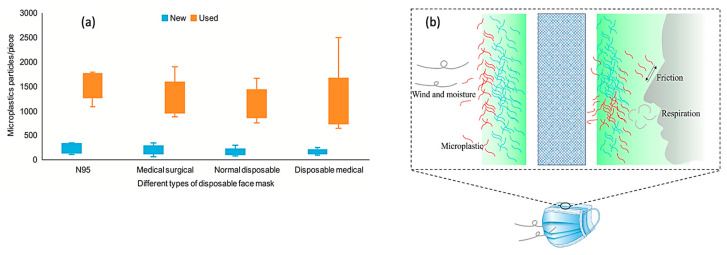
(**a**) The quantity difference between a new and used mask, and (**b**) its proposed mechanism diagram for the difference. Adapted with permission from Ref. [24]. Copyright 2021, Elsevier.

To comprehensively examine both the pathways and quantities associated with the release of plastic particles from masks into various environmental media, some scientists commonly obtain their findings by leaching masks to processes within a laboratory beaker or similar glassware. Some researchers have measured the possible amount of microplastics released into the aquatic environment by mixing masks with deionized water in a glass Erlenmeyer flask before shaking them for 24 h on a rotary shaker at a certain speed [24]. The samples were then rinsed with deionized water to collect all the microplastics that were adsorbed onto the surface of the masks. The initial deionized water and the rinsing one were eventually passed through a membrane filter to collect the microplastics, with a stereomicroscope subsequently used to determine the amount which had been released into the water. Within 24 h, an average of 183.00 ± 78.42 pieces of microplastics were obtained from new face masks compared with the 1246.62 ± 403.50 pieces released from used ones (Figure 2a). This difference between the new and used masks could be attributed to the fact that friction between the face and inner layer mask accelerated the release of microplastics from the mask. In addition, wind and moisture in the atmosphere could also have broken the outer layer of the masks (Figure 2b). In undertaking an investigation on the dynamic release of microplastics from face masks into an aqueous solution [25], such studies further indicated that the type of mask had little influence on the release kinetics of microplastics. In addition, microplastics measuring less than 500 μm had a higher release rate and were present in larger amounts compared with those of more than 500 μm in size. This difference was probably due to the fact that longer or larger microplastics need to be decomposed into smaller ones, with the process taking longer. Furthermore, natural weathering may also affect the release of microplastics from the masks, with old ones releasing almost 2.5 × 10^4^ times more microplastics compared with fresh masks [26]. However, as noted before, the brand or price of the masks had little correlation to the release quantity of microplastics into an aqueous solution. Finally, microplastics were usually quickly released on the first day, with the release rate being lower on subsequent days [25].

To further analyze the release characteristics of microplastics, the amount and morphology of microplastics released from different types of disposable face masks under laboratory conditions are listed in Table 1. According to these very recent reports, it can be concluded that the types of masks (e.g., N95 masks, surgical masks, and normal medical masks) do not significantly affect the release of microplastics under the same experimental conditions. However, the released amounts of microplastics have significant differences in different reports, which is mainly due to the differences in the application environment, degree of use, experimental conditions, etc. of the used masks obtained by different researchers. For example, it is known that masks undergo wear and aging during use, significantly increasing the release quantity and kinetics of microplastics. Therefore, under similar conditions, the amount of microplastics released from masks after prolonged use is significantly higher than that from new masks. Moreover, the specific layers of the masks play a crucial role in determining the extent of microplastic release. The inner and outer layers, being in frequent contact with the external environment, exhibit a higher propensity to release microplastics compared to the middle layer [24]. This heightened release can be attributed to the structural damage incurred and the material’s wear and aging over time. As these layers experience constant interaction with external factors such as air, moisture, and friction, the microplastics embedded in the mask material are more readily released into the surroundings. In summary, the above experimental results illustrate that the release characteristics of microplastics are possibly affected by multiple factors such as environmental conditions, test conditions, water conditions, and the degree of use [24,25,26,27,28]. The dynamic interplay of these factors underscores the nuanced nature of microplastic release, reinforcing the notion that it is a multifaceted phenomenon susceptible to diverse influences.

## 3. The Morphology of Microplastics Derived from Face Masks

The microplastics released from used masks would actually pose a threat to the air, water and soil environment [24,30]. During the COVID-19 pandemic periods, a significantly higher number of used masks were observed near lakes or rivers [31], resulting in more microplastics being released into the natural environment. In particular, along coastal areas, the microfibers (mainly those composed of polypropylene) could be released from broken face masks due to the rush of water [32]. In the context of face masks, the issue of microplastic pollution becomes especially pronounced, considering the widespread use and improper disposal of these essential protective equipment.

**Figure 3:**
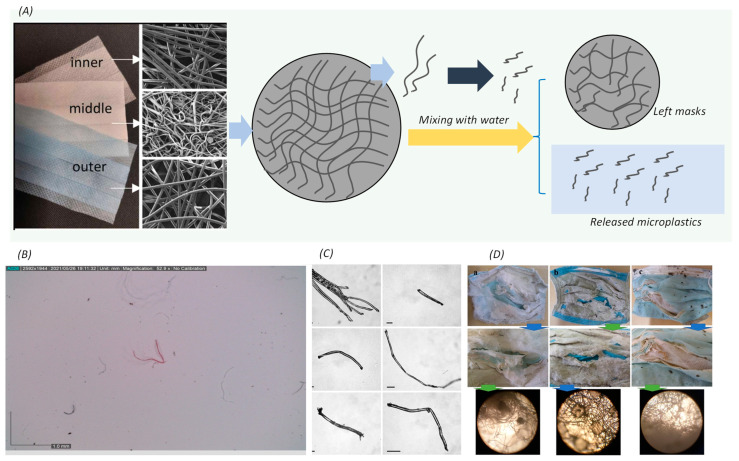
The morphology of plastic particles derived from the used face masks: (**A**) the proposed release mechanism of microplastics from masks. Adapted with permission from Ref. [40]. Copyright 2022, Elsevier; (**B**,**C**) at the shear experimental condition in the water environment [34]. Adapted with permission from Ref. [14]. Copyright 2021, Elsevier; and (**D**) collected from the natural seaside. Adapted with permission from Ref. [32]. Copyright 2021, Elsevier.

Generally, the plastic pollutants released from the used face masks are present in the form of polymeric fibers, particles and fragments that might have unique properties and implications for environmental fate and human health [33]. In the aquatic environment, the chemical bond of the plastic fibers of the face masks would be broken under the force of the water flow, thus releasing some broken fibers and fragmented particles into the aqueous surroundings, becoming part of the aqueous environment (Figure 3A). Under the experimental conditions for masks in an aqueous environment, the released plastics were commonly presenting a fiber-like shape with an average size of 1.7 mm [34], while other studies have found that the released fibers were of less than 3 mm in size (Figure 3B,C) [28]. Herein, the size of the plastic fibers or particles released from the masks was generally maintained at the millimeter scale. The plastic fibers or particles within the above size range were more likely to adsorb other pollutants in the aquatic environment. For instance, along the natural seaside, the layer structure of disposable face masks was easily destroyed by the water flow, resulting in the generation of microfiber-like plastics that subsequently flowed into the ocean (Figure 3D). Moreover, weathering effects would play a crucial role in accelerating the release of microplastics from the used masks [35,36,37]. Especially under the influence of the UV weathering process, the polymer components of masks would undergo a relatively complex process, where the processes of scission and crosslinking may occur simultaneously. The scission process was primarily achieved through the breaking of molecular bonds of the polymer components, while the crosslinking process would commonly involve the interaction between the polymers (such as polyethylene, polypropylene, and polystyrene) and certain monomers or functional groups [38,39]. Herein, in a short-term UV weathering process, most of the released particle sizes fall within the size distribution of less than 200 µm. However, as the duration of UV weathering increases, there is a corresponding increase in particle size [35].

## 4. Potential Interactions between the Microplastics (from Masks) and Other Pollutants

**Figure 4:**
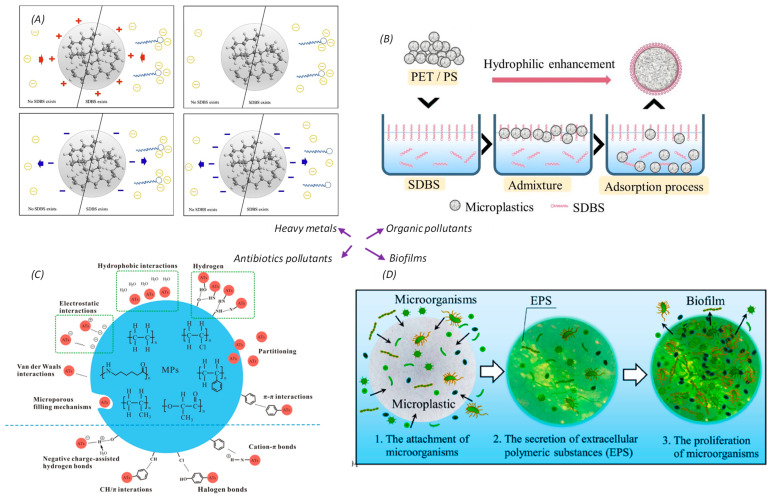
The potential interaction mechanism between microplastics and other pollutants in the water environment: (**A**) microplastics with heavy metals. Adapted with permission from Ref. [15]. Copyright 2020, Elsevier; (**B**) PET (Polyethylene terephthalate) and PS (Polystyrene) microplastics interfacial contact with organic pollutants of sodium dodecyl benzene sulfonate (SDBS). Adapted with permission from Ref. [18]. Copyright 2021, Elsevier; (**C**) microplastics with antibiotics pollutants. Adapted with permission from Ref. [42]. Copyright 2021, ACS Publications; (**D**) microplastics with biofilm. Adapted with permission from Ref. [17]. Copyright 2021, ACS Publications.

Recently, many research groups have investigated possible interactions between microplastics and other pollutants, such as heavy metals, organic pollutants or even biofilms, in the aquatic environment [15,17,18,41,42,43,44]. Due to the presence of various types of pollutants, potential interactions between microplastics and other particles would also be different. For instance, in aqueous environments, possible interactions between heavy metal ions and microplastics mainly involved electrostatic attraction (or repulsion) as well as hydrophobic interactions [15]. In addition, the presence of additives such as surfactants in the water may also affect the adsorption of heavy metal ions onto the microplastics (Figure 4A). On the other hand, the potential contact force between SDBS (sodium dodecyl benzene sulfonate, a typical organic pollutant) and microplastics mainly include hydrophobic interactions, ion-pair adsorption, ion exchange adsorption, and dispersive force adsorption [18], with the hydrophobic one being the most important (Figure 4B). Similarly, interactions between microplastics and antibiotic pollutants (one type of emerging pollutants) are mainly due to hydrophobic interactions, electrostatic interactions, as well as hydrogen bonding (Figure 4C) [42]. In the natural aquatic environment, biofilms also tend to be formed on the surface of microplastics. During the formation of such biofilms (Figure 4D), microorganisms first attach on the microplastics, and after secreting EPS (extracellular polymeric substances), they start to proliferate [17].

The microplastics released from face masks commonly display fiber shapes, especially in the case of finer particles. The adsorption ratio (or capacity) of pollutants onto fiber microplastics (released from the face masks) can be significantly different from that of more common types of microplastics. This distinctive morphology raises intriguing questions about the adsorption dynamics of pollutants onto fiber microplastics, specifically those emanating from face masks. 

Distinguishing the unique properties of microplastics originating from face masks was pivotal in understanding their environmental impact. Compared with conventional microplastics, fiber shaped microplastics presented a certain process complexity when contacting other pollutants. The presence of similar plastic components in fiber microplastics and their round counterparts suggests that commonalities may exist in the mechanisms governing pollutant adsorption. This conclusion opens avenues for comparative analyses, exploring whether lessons learned from the broader microplastics field can be applied to the specific context of face mask-derived microplastics.

## 5. Challenges for Control of Microplastic Release from Personal Protective Equipment (PPE)

In a comprehensive review of both domestic and international literature, it becomes apparent that the current state of research on the release of microplastics from PPE within the general population and societal contexts has predominantly centered on quantifying the release of plastic particles [43,44,45]. Notably, those investigations were mainly conducted on a laboratory simulation scale. However, a significant gap still persisted concerning the relationship between the quantities of microplastics released in simulated scenarios and their actual release into the environment. Bridging this gap will be essential for a comprehensive understanding of the environmental impact of microplastic release from PPE in the natural environment. Herein, we should further identify the primary modes and sources through which PPE releases microplastics into the natural environment. It is necessary to analyze the contributions of improper PPE waste disposal, casual littering by the public, emissions from industries related to PPE, and natural oceanic currents to the distribution of microplastics in natural environment. Through understanding the detailed sources of microplastics, we can implement corresponding measures to reduce the quantity of plastic particles released from PPE into the natural environment.

Aiming to achieve a more robust and scientifically informed assessment result, it is imperative to delve into the intricate dynamics of plastic particle release from PPE by the material flow analysis route [46,47,48]. This comprehensive analysis process is crucial for accurately estimating the global or regional scale of plastic particle release from PPE, taking into account different PPE usage scenarios and disposal methods. Through exploring the nuances of the release and disposal processes, researchers could gain insights into the potential environmental contamination stemming from microplastic release to environment. Herein, we should track their fate and understand their potential destiny of plastic particle derived from PPE in the natural environment.

Furthermore, it is imperative to delve into comprehensive research on the intricate interface interactions between the released plastic particles (from PPE) and pollutants. This investigation should encompass various perspectives, including biological [48,49,50], physicochemical [14,15], and other relevant angles [51]. Such a multifaceted approach is essential for unraveling the complex network of ecological and toxicological impacts that are intricately linked with microplastic particle pollution. A thorough understanding of how the released microplastic particles interact with pollutants is pivotal for scientists in accurately assessing the potential risks posed to both ecosystems and human health. This exploration would provide a foundation for developing effective strategies to mitigate the adverse effects of plastic particle pollution on the environment and biological systems, ensuring a more sustainable and healthier future.

In conclusion, it is urgent to address key gaps in our current understanding of microplastic release from PPE. A more holistic and interdisciplinary approach, integrating findings from both laboratory simulations and real-world environmental observations, is paramount for advancing our knowledge and devising effective strategies to mitigate the environmental impact of plastic particle release from PPE. In the broader context of environmental sustainability, understanding the intricate dynamics of microplastic release from masks becomes imperative. It necessitates a holistic approach that considers not only the immediate benefits of mask usage for public health but also the long-term consequences for our ecosystems. Striking a balance between effective disease prevention measures and minimizing the environmental footprint of essential protective equipment like masks requires thoughtful consideration and the implementation of sustainable practices. As we navigate these challenges, it becomes crucial to develop innovative solutions and waste management strategies that address both the public health imperative and environmental conservation goals.

## 6. Conclusions

This paper mainly focuses on reviewing pollution caused by microplastics (released from used face masks) in the aquatic environment. It introduces an experiment-based approach that has been used to determine the amount and morphology of fiber microplastics released from face masks into the aqueous surrounding. Finally, the possible interactions between microplastics and other pollutants in an aqueous environment were also reviewed.

(1) The amount of microplastics released from used face masks into water can be affected by multiple factors, such as test conditions, water quality, and degree of use, etc. The average amount of microplastics released from the masks ranged from 47 to more than 10,000 pieces per day for each mask under different environmental conditions. Furthermore, microplastic pollutants were mainly present in the form of polymeric fibers, with some finer particles. Within aquatic environments, the chemical bonds of the plastic fibers are also destroyed by the force of water flow, resulting in the generation of smaller fiber microplastics.
(2) The dominant interface forces between the microplastics and other pollutants in an aqueous environment mainly involve hydrophobic interactions, electrostatic interactions as well as hydrogen bonding. To some extent, the shape of the fiber microplastics, released from face masks also tends to be different from the round microplastics commonly observed in the natural environment. However, the possible contact force between the pollutants and the microplastics largely remains as hydrophobic and electrostatic interactions. Thus, the fiber microplastics released from used face masks would have a tendency to adsorb other pollutants including heavy metals, organic pollutants or even biofilms.
(3) In anticipation of forthcoming issues related to microplastic pollution arising from PPE, it is imperative to identify the primary sources and pathways through which microplastics from PPE enter the natural environment. Utilizing methods such as material flow analysis or more precise analytical routes is essential for gaining a comprehensive understanding of the flow of microplastics. Additionally, conducting thorough research on the intricate interface interactions between the plastic particles released from PPE and pollutants is crucial. This research aims to predict the potential risks posed to both ecosystems and human health, thereby enhancing our ability to proactively address and mitigate the impacts of microplastic pollution. Based on a systematic analysis of the origin, entire process, and interface interactions of plastic particles (from PPE) in natural environment, it is crucial to facilitate the optimal selection of relevant management or technological approaches for the effective control and treatment of microplastics.

## Figures and Tables

**Table 1:** The quantity and morphology of microplastics released from the used face masks.

Index	Type of Mask	Microplastics Release/Piece·Day^−1^	Microplastics Morphology	Ref.
1	Disposable face mask	1246.62 ± 403.50	fiber	[24]
2	N95 Mask	801 ± 71–2667 ± 97	fiber	[25]
	medical surgical mask	1136 ± 87–2343 ± 168		
	Normal medical mask	1034 ± 119–2547 ± 185		
3	Disposable surgical mask	116,600 (in water) 168,800 (in detergent) 147,000 (in alcohol)	fiber	[26]
4	Surgical face mask	173,000 (in seawater)	fiber	[27]
5	medical surface mask	1447 ± 218	Fiber/particle	[29]
	N95 mask	1339 ± 166		
	activated carbon mask	1600 ± 237		

## Data Availability

No new data were created or analyzed in this study. Data sharing is not applicable to this article.
